# Effects of HH education program on HH compliance among empolyess working in a tertiary care setting in Saudi Arabia

**DOI:** 10.1186/1753-6561-5-S6-P127

**Published:** 2011-06-29

**Authors:** HH Balkhy, E Tannous, A El-Saed

**Affiliations:** 1 Infection Prevention and Control Department, KAMC, Riyadh, Saudi Arabia

## Introduction / objectives

King Abdulaziz Medical City conducted a pilot study monitoring the rates of HH compliance among healthcare workers over time.

## Methods

Two phases were identified. The **pilot phase,** where HH compliance was monitored in adult ICUs during a baseline period and again during a follow up period; and the **implementation phase** where monthly and quarterly feedback of HH compliance was provided.

## Results

A total of 1,658 HH opportunities were observed during the pilot phase and more than 55,000 opportunities were monitored over the next 2 years of implementation. Compared to baseline, rubbing increased (63.8% to 85.0%) while washing decreased (36.2 to 15.0%) during the follow up period (p<0.001). Baseline HH compliance rate was 45.3% and 76.1% thereafter. With an absolute improvement of 30.8% and relative improvement of 68.0%. The absolute improvement of compliance rate (average 30.8) was highest among nurses (34.6%) followed by other HCWs (28.0%) and finally doctors (21.9%). All HH indications had significant improvements with the exception of HH after body fluid exposure.

## Conclusion

The initial improvement observed at the pilot phase of HH program at KAMC was maintained and actually improved.

## Disclosure of interest

None declared.

